# Extension of Sylvatic Circulation of African Swine Fever Virus in Extralimital Warthogs in South Africa

**DOI:** 10.3389/fvets.2021.746129

**Published:** 2021-11-24

**Authors:** Anthony F. Craig, Mathilde L. Schade-Weskott, Henry J. Harris, Livio Heath, Gideon J. P. Kriel, Lin-Mari de Klerk-Lorist, Louis van Schalkwyk, Peter Buss, Jessie D. Trujillo, Jan E. Crafford, Juergen A. Richt, Robert Swanepoel

**Affiliations:** ^1^Vectors and Vector-Borne Diseases Research Programme, Department of Veterinary Tropical Diseases, Faculty of Veterinary Science, University of Pretoria, Pretoria, South Africa; ^2^Agricultural Research Council-Onderstepoort Veterinary Research Transboundary Animal Diseases Laboratory, Pretoria, South Africa; ^3^Provincial Veterinary Services, Department of Agriculture, Land Reform and Rural Development, Kimberley, South Africa; ^4^Office of the State Veterinarian, Department of Agriculture, Land Reform and Rural Development, Kruger National Park, Skukuza, South Africa; ^5^Department of Migration, Max Planck Institute of Animal Behavior, Radolfzell, Germany; ^6^Veterinary Wildlife Services, South African National Parks, Kruger National Park, Skukuza, South Africa; ^7^Diagnostic Medicine/Pathobiology, Center of Excellence for Emerging and Zoonotic Animal Diseases (CEEZAD), College of Veterinary Medicine, Kansas State University, Manhattan, KS, United States

**Keywords:** African swine fever virus (ASFV), sylvatic circulation, extralimital warthogs, South Africa, serosurveilance

## Abstract

Sylvatic circulation of African swine fever virus (ASFV) in warthogs and *Ornithodoros* ticks that live in warthog burrows historically occurred in northern South Africa. Outbreaks of the disease in domestic pigs originated in this region. A controlled area was declared in the north in 1935 and regulations were implemented to prevent transfer of potentially infected suids or products to the rest of the country. However, over the past six decades, warthogs have been widely translocated to the south where the extralimital animals have flourished to become an invasive species. Since 2016, there have been outbreaks of ASF in pigs outside the controlled area that cannot be linked to transfer of infected animals or products from the north. An investigation in 2008–2012 revealed that the presence of *Ornithodoros* ticks and ASFV in warthog burrows extended marginally across the boundary of the controlled area. We found serological evidence of ASFV circulation in extralimital warthogs further south in the central part of the country.

## Introduction

African swine fever virus (ASFV) was first recognized in 1910 as the causative agent of a contagious and lethal disease of domestic pigs introduced into Kenya during the colonial era and farmed in proximity to wild suids (1). It subsequently emerged that the virus was maintained in the savannah areas of eastern and southern Africa through sylvatic circulation in the common warthog (*Phacochoerus africanus*) that does not become ill after infection and eyeless argasid ticks of the *Ornithodoros (Ornithodoros) moubata* complex that live in warthog burrows (2–5). More importantly, the virus can also be maintained through uncontrolled spread in populations of domestic pigs, and this has been facilitated in recent decades by widespread increase in small-scale pig farming in Africa that frequently involves free-ranging animals and informal trading in communal and peri-urban areas (6, 7).

**Figure 1:**
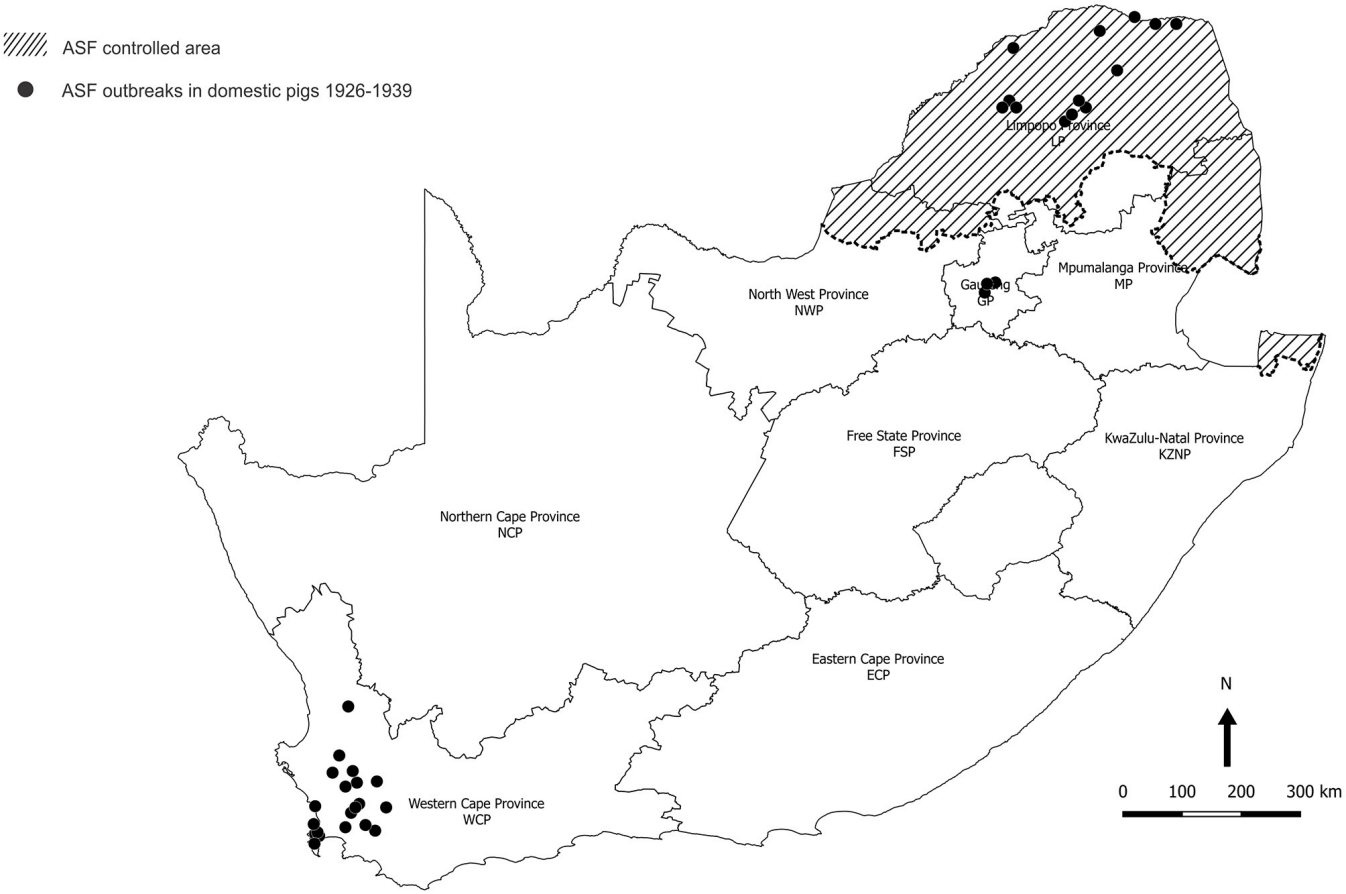
Distribution of 37 outbreaks of African swine fever in South Africa, 1926–1939, for which approximate coordinates (correct to 0.1 degree) were derived from references cited in the text. The South African Protection of Personal Information Act 4 of 2013 precludes divulging names and accurate coordinates of private property.

In South Africa, the disease was first recognized in 1926 in pigs farmed in the northernmost Limpopo Province (LP) where warthogs were present (8, 9). Outbreaks of the disease either occurred in the north of the country or were initiated elsewhere through movement of infected pigs or pork products from the north, notably to farms in the environs of Johannesburg in Gauteng Province (GP) and from there to properties in Western Cape Province (WCP) (10). Consequently, an ASF controlled area was declared in 1935 to include the known distribution range of warthogs at that time (Figure 1) (10). Regulations were instituted to prevent movement of infected pigs or products from the controlled area, and to ensure that outbreaks were eradicated through slaughter of infected herds plus disinfection and quarantine of premises. Carcasses of hunted or culled wild suids could be transported under veterinary permit provided that the skin, head, hooves, and internal organs were removed (10).

**Figure 2:**
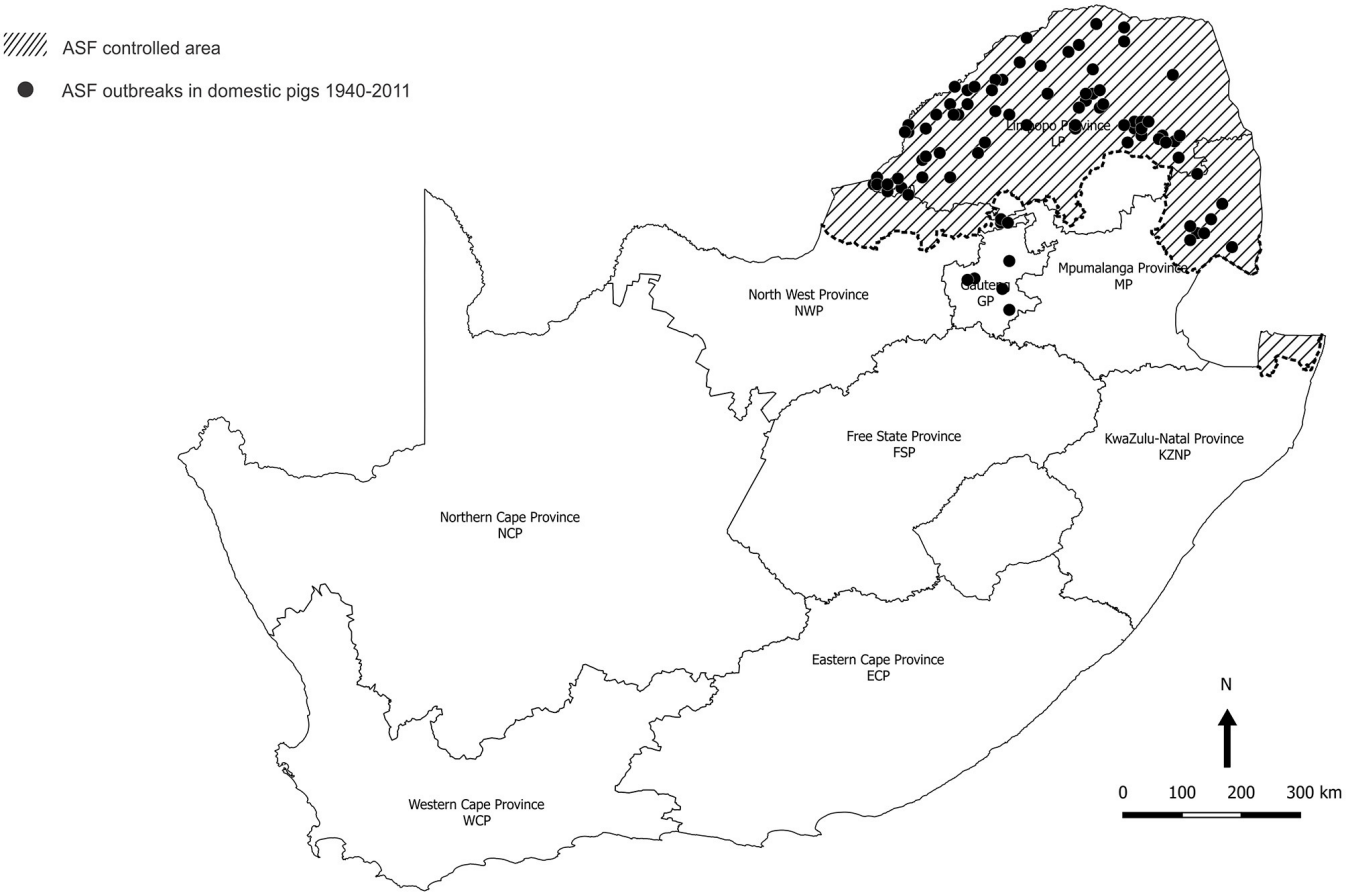
Distribution of 89 outbreaks of African swine fever in South Africa, 1940–2011, for which approximate coordinates (correct to 0.1 degree) were derived from references cited in the text.

**Figure 3:**
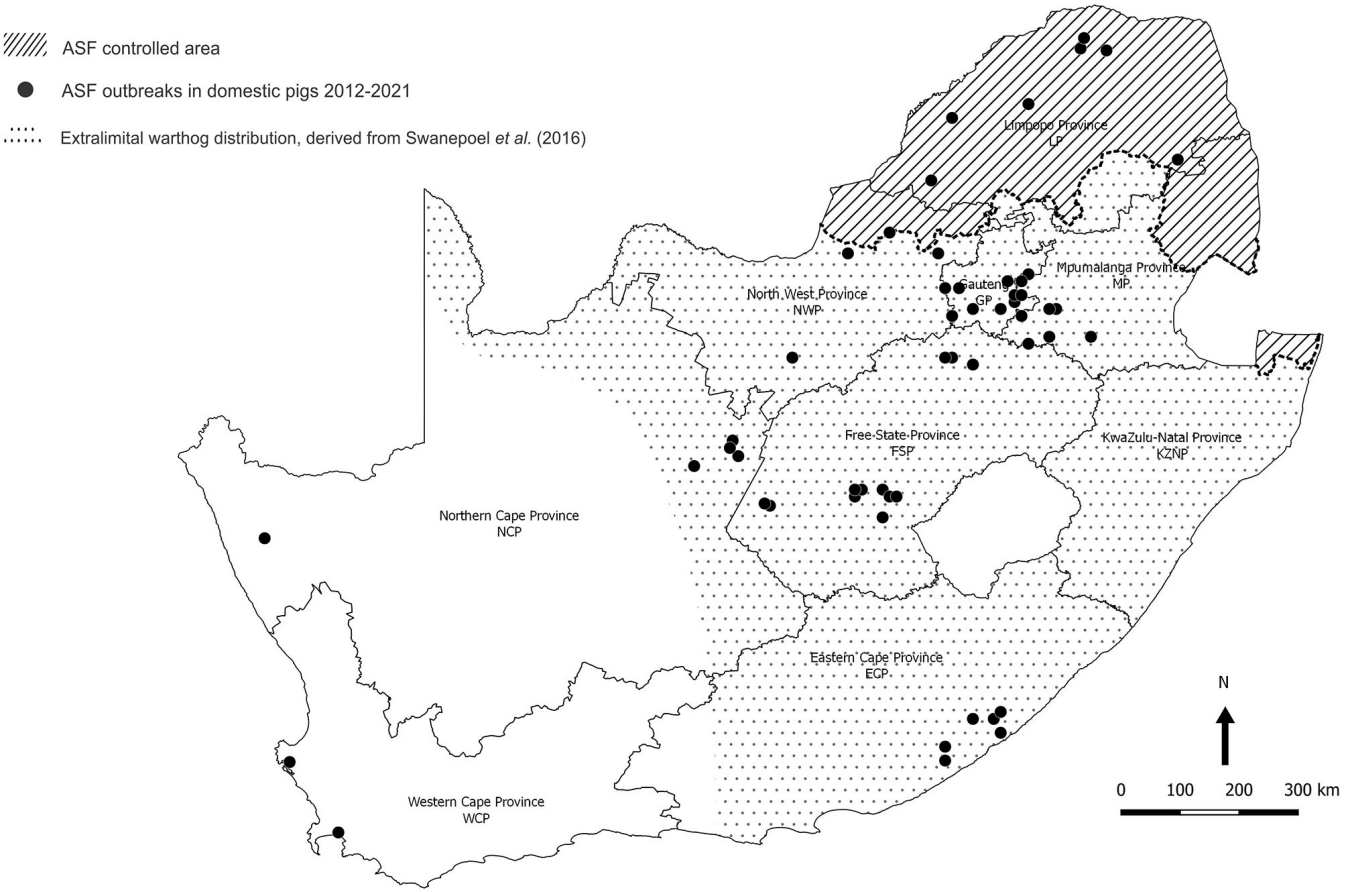
Distribution of 85 outbreaks of African swine fever in South Africa, 2012–2020, for which approximate coordinates (correct to 0.1 degree) were derived from references cited in the text. The distribution of warthogs outside the controlled area was plotted with QGIS (Penn Libraries, Philadelphia, PA) using data from Swanepoel et al. (17).

The last outbreak in WCP was only eradicated in 1939. There were a few outbreaks of ASF in 1951 associated with the feeding of swill from five local abattoirs in GP, one of which is known to have received a consignment of infected pigs from Namibia (11). In 1996, isolated outbreaks occurred on three adjoining farms in northern GP close to the boundary of the controlled area (Figure 2) (12). Thereafter, the regulatory measures proved effective until 2012 when a series of outbreaks on smallholdings in adjacent areas of GP and Mpumalanga Province (MP) were traced to initial illegal movement of infected pigs from LP followed by local spread of infection involving sale of animals at auctions (12–14). The virus genotype involved, XXII, had previously been detected in the district of origin of the infected pigs in LP (15, 16). From 2016 onwards, there have been successive series of outbreaks of ASF in domestic pigs in GP, MP, North West Province (NWP), Northern Cape Province (NCP), and Free State Province (FSP) that cannot be linked to movement of infected pigs or products from the controlled area (Figure 3) (15, 18–20). A structured serosurvey found that the outbreaks of 2016 and 2017 had been eradicated effectively, with no evidence of persistence of infection in local pig populations (21).

Meanwhile, from 1963 onwards, there had been widespread translocation of warthogs to the south of the country associated with the growth of an extensive wildlife ranching and conservation industry, and the extralimital animals flourished to the extent that they are regarded as an invasive species (Figure 3) (17, 22–24). An investigation conducted in 2008–2012 on farms within 20 km of the boundary revealed the presence of *Ornithodoros* ticks in warthog burrows beyond the controlled area in NWP, GP, LP, and MP, with ASFV detected in one pool of ticks from MP (14). We were prompted to test warthog sera acquired opportunistically for evidence of sylvatic circulation of ASFV further south, and the findings are presented here.

## Materials and Methods

### Study Sites

**Figure 4:**
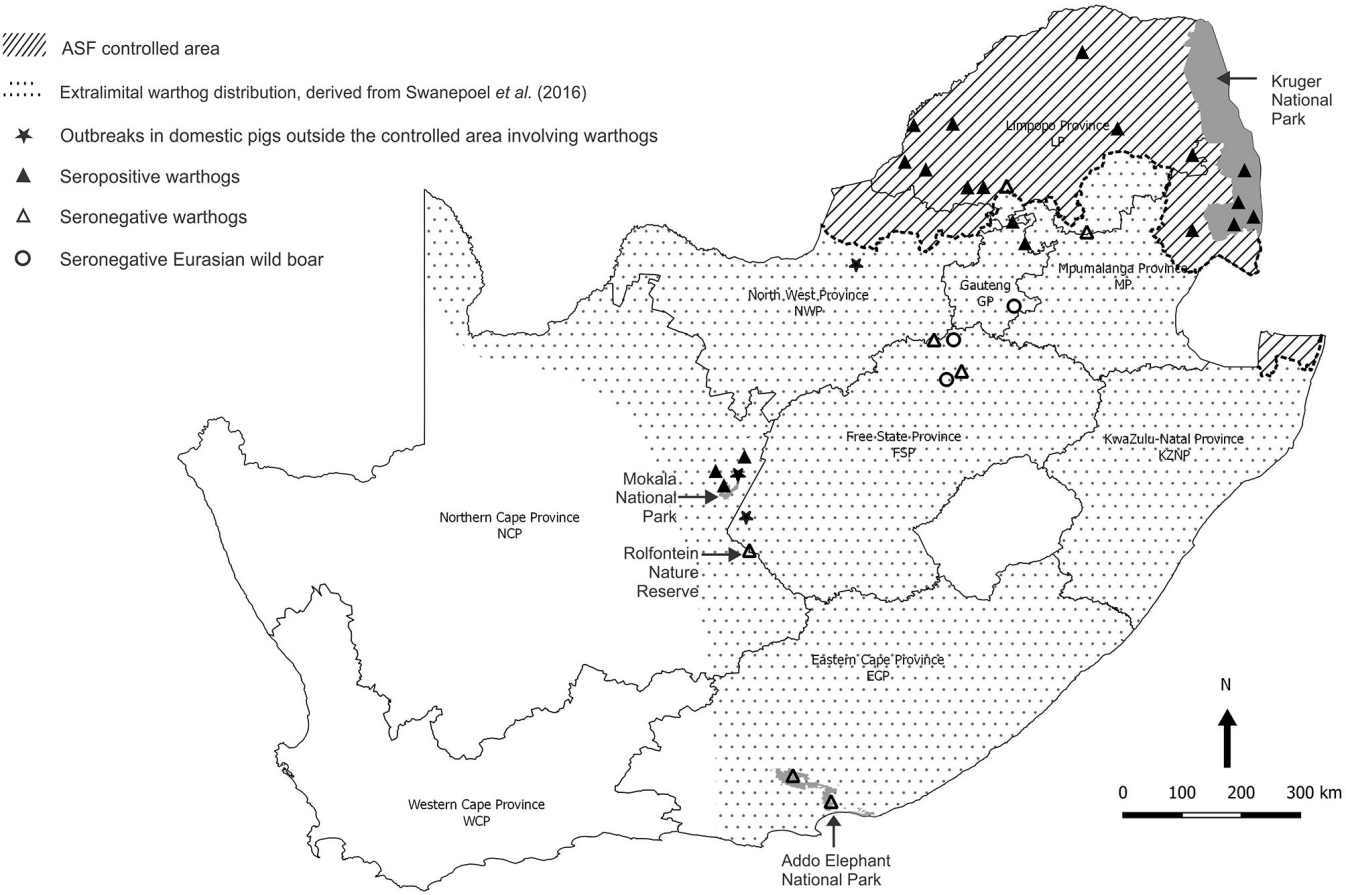
Sites where samples from warthogs and Eurasian wild boars were obtained and tested for evidence of infection with African swine fever virus, and farms outside the controlled area where African swine fever outbreaks in domestic pigs were ascribed to contact with warthogs in 2017 and 2019.

Baseline observations were made on serum samples obtained from warthogs in the Greater Kruger National Park (GKNP) that comprises the national park plus 20 adjoining private parks and lies within the controlled area where ASFV is known to be prevalent (Figure 4). For contrast, serum samples were collected in 2019 during a warthog culling operation in Addo Elephant National Park (Addo ENP) in ECP where ASF had never been recorded. Specific evidence of sylvatic circulation of ASFV beyond the controlled area was sought in the NCP by testing samples collected during a warthog culling operation in 2019 in Mokala National Park (Mokala NP), plus stored serum samples that had been collected earlier in the same park and in the provincial Rolfontein Nature Reserve (Rolfontein NR) that lies south of Mokala NP (Figure 4). In addition, blood samples from wild suids hunted or culled on private properties were sought through negotiation with organizations representing the wildlife industry, or collected in association with provincial veterinary officials in the vicinity of past outbreaks of ASF (Figure 4).

### Samples

Altogether, 2,469 samples were collected from 546 warthogs in one provincial and three national nature reserves, including 783 duplicate tissue samples in formalin-fixative (Table 1).

**Table 1:** Samples obtained from warthogs in four nature reserves in South Africa (abbreviations as given in the text).

	**GKNP**	**Addo ENP**	**Mokala NP**		**Rolfontein NR**
**Years of sampling**	**1999–2020**	**2019**	**2017**	**2019**	**2016**
Serum	207	116	23	77	44
EDTA blood	3	114	0	75	0
Spleen	11	36	0	82	0
Miscellaneous organs	14	0	0	0	0
Peripheral LN	95	137	0	82	0
Mediastinal LN	49	134	0	82	0
Mesenteric LN	41	14	0	81	0
Bone marrow	34	50	0	81	0
Fetal thymus	0	0	0	2	0
Fetal adrenal	0	0	0	2	0
Sub-total	454	601	23	564	44
Formalin-fixed duplicates	0	371	0	412	0
Total samples	454	972	999		44
Total warthogs sampled	241	154	107		44

Samples from GKNP consisted mainly of serum collected from warthogs translocated internally or culled for managerial purposes from 1999 to 2020 and stored at −80°C, but included a few blood samples and a number of lymph node and visceral organ samples collected from culled animals for an unrelated study (25) (Table 1).

Samples collected during the culling operations in Addo ENP and Mokala NP in 2019 comprised clotted blood for serum, whole blood collected with EDTA, mandibular, mediastinal, and mesenteric lymph nodes, plus spleen and bone marrow. Thymus and adrenal samples were collected from two fetuses encountered in Mokala NP. Samples other than blood or serum were collected mainly for a subsidiary study on distribution of ASFV in warthog tissues to be reported separately. A serial number was allocated to each warthog, and the date of sampling, gender, and age group estimated from size and dentition (26) were recorded. Access was obtained retrospectively to 23 warthog sera that had been collected during a culling operation in 2017 in Mokala NP and stored at −80°C, plus 44 warthog sera collected in 2016 and stored at −20°C in the provincial Rolfontein NR situated approximately 100 km south of Mokala NP.

Blood samples from animals hunted or culled on private properties were intended to include any wild suids that may be encountered, not just warthogs. In the event, only 48 samples were received from 19 properties (farms or small nature reserves), 10 of which were situated inside the controlled area and 9 outside (Table 2). The samples comprised dried blood collected on Nobuto cellulose strips (NCS) (Advantec, Tokyo, Japan) or clotted blood from warthogs and Eurasian wild boars submitted by landowners and hunters from properties 1 to 17, plus clotted blood, mediastinal lymph node, and spleen samples from 4 warthogs collected in association with provincial veterinary officials during investigations on properties 18 and 19 in the vicinity of past ASF outbreaks in NCP (Table 2).

**Table 2:** Dried blood samples on Nobuto cellulose strips (Ncs) and clotted blood samples from warthogs or Eurasian wild boars received from hunters or landowners from 10 properties inside and 9 outside the African swine fever controlled area in South Africa during 2019 and 2020 (abbreviations as given in the text).

**Property**	**Municipal area**	**Province**	**Species**	**Sample**	**No. of samples**	**ASF ELISA positive**
**Properties inside the ASF controlled area**						
1	Makhado	LP	Warthog	Ncs	3	3
2	Lephalale	LP	Warthog	Ncs	4	4
3	Lephalale	LP	Warthog	Ncs	3	3
4	Thabazimbi	LP	Warthog	Ncs	4	4
5	Thabazimbi	LP	Warthog	Ncs	2	2
6	Thabazimbi	LP	Warthog	Ncs	3	3
7	Modimolle	LP	Warthog	Ncs	1	0
8	Bela Bela	LP	Warthog	Ncs	1	1
9	Bela Bela	LP	Warthog	Ncs	4	4
10	Mbombela	MP	Warthog	Ncs	4	2
**Properties outside the ASF controlled area**						
11	Bela Bela	LP	Warthog	Ncs	2	2
12	Elias Motsoaledi	LP	Warthog	Ncs	1	0
13	Tlokwe	NWP	Eu wild boar	Blood	1	0
13	Tlokwe	NWP	Warthog	Blood	1	0
14	Ekurhuleni	GP	Eu wild boar	Blood	1	0
15	Tshwane	GP	Warthog	Ncs	2	2
16	Ngwathe	FSP	Warthog	Ncs	2	0
17	Ngwathe	FSP	Eu wild boar	Ncs	1	0
18	Dikgatlong	NCP	Warthog	Blood^*^	2	2
19	Dikgatlong	NCP	Warthog	Blood^*^	2	2
					44	34

* *Additionally, mediastinal lymph node and spleen samples were obtained from the same animals*.

### Detection of Antibody to ASFV

Tests for antibody to ASFV p72 protein in serum samples were performed with INgezim PPA Compac R.11.PPA.K3 blocking enzyme-linked immunosorbent assay (ELISA) kits (Eurofins Technologies Ingenasa, Madrid, Spain) used according to the manufacturer's instructions. Dried blood samples on NCS were eluted with kit buffer before testing (27).

### Nucleic Acid Extraction, ASFV DNA Detection, and p72 Genotyping

Approximately 10% (w/v) suspensions of warthog tissues were prepared by homogenizing samples in phosphate-buffered saline, pH 7.2 (PBS). Automated nucleic acid extraction was performed with IndiMag Pathogen kits (Indical Bioscience, Leipzig, Germany) using slight modifications to the manufacturer's instructions. Briefly, 200 μl of tissue homogenate supernatant was added to 300 μl of ATL buffer (Qiagen, Dusseldorf, Germany) and 40 μl of proteinase K (Qiagen) and incubated at 56°C for 2 h before 200 μl of the lysed sample was added to 200 μl of AL buffer (Qiagen) and incubated at 70°C for 10 min. For whole blood samples, 200 μl was added directly to 200 μl of AL buffer and mixed well. For all samples, 200 μl of the AL lysate was added to the IndiMag buffer for extraction. Each extraction included known ASFV-positive controls. Eluates were stored at −80°C until further use.

Eluates were tested for ASFV nucleic acid using the real-time quantitative PCR (qPCR) assay of Zsak et al. (28) with modifications (29). Briefly, 5 μl of DNA was amplified in 20-μl reactions using 20 pmol of the published primers and 7 pmol of probe in Perfecta Fastmix II (Quanta Biosciences, Beverly, MA). Positive and no template controls (NTC) were included for each PCR run. Samples with Cq mean values ≤ 38 (selected as the cutoff value based on the analytical sensitivity limits of the qPCR assay) were considered positive.

To confirm that ASFV was identified in warthog tissues from Mokala NP, nucleic acid from the qPCR-positive spleen sample with lowest Cq mean value was amplified using primers p72-D and p72-U and the cycling conditions of Bastos et al. (30). An appropriately sized band of amplification product was excised from the agarose electrophoresis gel, purified, and subjected to Sanger nucleotide sequencing. The sequence was viewed and maximum likelihood phylogenetic comparisons made with representative sequences of the 24 known ASFV p72 genotypes (31) using MEGA X software (32).

## Results

**Figure 5:**
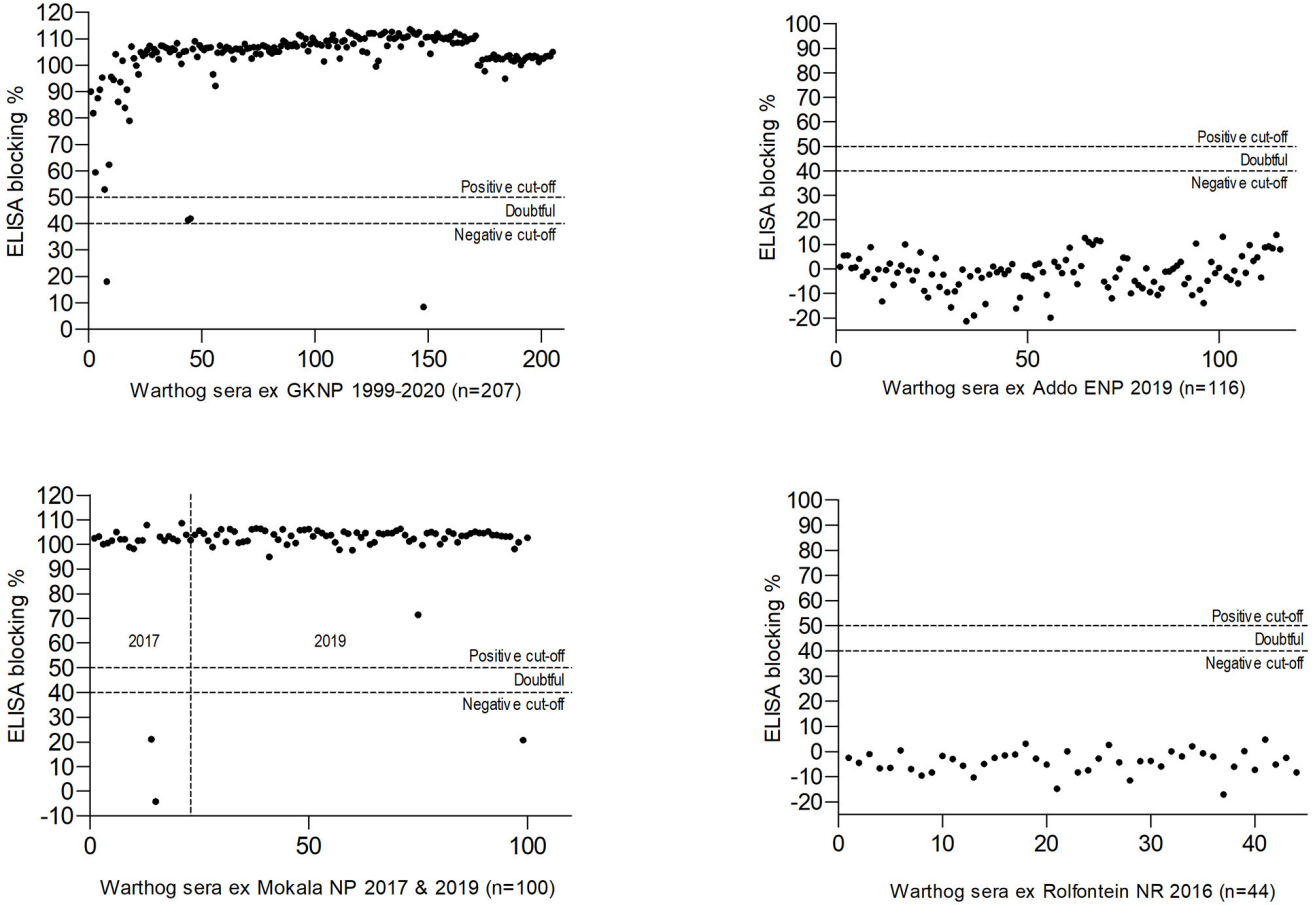
Results of blocking enzyme-linked immunosorbent assays (ELISA) for antibody to African swine fever virus performed on warthog sera obtained from four nature reserves in South Africa (abbreviations as given in the text).

The results obtained in the ELISA for antibody to ASFV in warthog sera from the four nature reserves are presented in Figures 4, 5. As expected, a high prevalence of antibody was found in the sera of warthogs from GKNP, 97.1% (201/207), with doubtful reactions recorded in a further two juveniles, while no antibody activity was detected in sera from Addo ENP in ECP where no ASF outbreaks had ever been recorded. In contrast, a high prevalence of antibody was detected in sera collected during the 2019 culling operation in Mokala NP, 98.7% (76/77), some 400 km south of the controlled area, and also in the sera collected there in 2017, 91.3% (21/23), while no antibody was detected in the 44 samples collected in Rolfontein NR in 2016.

A high prevalence of antibody, 26/29 (89.6%), was detected in dried warthog blood samples obtained from 9/10 private properties within the ASF controlled area (Table 2 and Figure 4). Outside the controlled area, antibody was detected on only 4/9 properties. These included positive reactions in four warthog blood samples from properties 11 and 15 (a small nature reserve), that lie close to each other and to the three farms where ASF outbreaks occurred in 1996 within 40 km south of the boundary of the controlled area near Pretoria in northern GP, plus four warthog sera from properties 18 and 19 that are situated about 100 km north and west of Mokala NP in the vicinity of past outbreaks of ASF in pigs in NCP. The remaining samples tested negative despite the fact that two sera from properties 13 and 17 in NWP and FSP came from Eurasian wild boars shot as free-ranging animals in proximity to free-ranging warthogs. In the course of the investigations, histories were obtained to confirm that fresh warthog offal had been fed to pigs on one farm where an outbreak of ASF had occurred in NCP in 2017, and that free-roaming pigs had been in contact with warthogs, including carcasses, on a farm where an outbreak occurred in the adjacent eastern FSP in 2017, with both locations being over 100 km distant from Mokala NP (Table 2 and Figure 4) (20). In addition, an outbreak of ASF in 2019 involved both pigs and Eurasian wild boars that had escaped their pens on a farm where warthogs were present 10 km south of the controlled area in NWP (Table 2 and Figure 4) (20).

ELISA is not accredited for detection of ASF antibody in warthog sera, and in order to confirm the presumptive evidence for presence of the virus in Mokala NP, all 75 warthog EDTA blood samples and the 412 other tissue samples collected in Mokala NP in 2019 were tested for ASFV nucleic acid by qPCR (28, 29). All EDTA blood samples tested negative but 13 of the other samples from Mokala NP, including mandibular, mediastinal, and mesenteric lymph nodes plus spleen tested positive. The sample with the lowest Cq mean value, spleen from warthog Mokala NP 77, was selected for PCR with the Bastos et al. (30) p72 primers and the product determined to belong to the p72 genotype I of ASFV as had been identified in the disease outbreaks outside the controlled area in 2016 and subsequently in the same districts (15). The more recent outbreaks of ASF that occurred in ECP and WCP in 2020 and 2021 subsequent to the present sampling (Figure 3) were associated with genotype II virus, also detected in an outbreak in NWP in 2019 on the boundary of the controlled area, but otherwise associated with Mozambique, Madagascar, Mauritius, Tanzania, Zambia, and Zimbabwe (6, 7, 20, 30, 33–35).

## Discussion

Bushpigs (*Potamochoerus larvatus*) are the only indigenous wild suids present in South Africa apart from warthogs (36). They are widely distributed in wooded areas and sleep in lairs. Consequently, they do not have the same exposure to *Ornithodoros* ticks in burrows as warthogs. Bushpigs do not become ill following experimental inoculation with ASFV but develop viremic infection and can be transiently contagious for domestic pigs (37–40). Natural infection of bushpigs with ASFV, presumed to involve environmental contamination, has been reported in East Africa with some evidence of transmission of virus to domestic pigs (41–44).

In South Africa, bushpigs were stated to be less commonly infected with ASFV than warthogs and have never been implicated in outbreaks of the disease in domestic pigs, although on occasion warthogs and Eurasian wild boars have been misidentified as bushpigs (16, 45). They are less frequently hunted than warthogs and no samples from bushpigs were received for testing during the present study.

Feral domestic pigs and Eurasian wild boars, *Sus scrofa ferus*, are exotic to sub-Saharan Africa. They are susceptible to the disease and can be involved in spread of infection by contagion. A recently discovered mechanism for maintenance of ASFV involves survival of infectivity in carcasses in the cold climate of northern Europe and scavenging by wild boars (7, 46).

Eurasian wild boars were introduced into South Africa to control pine tree moths in WCP where residual populations still exist, but apparently they did not adapt well to conditions in that region (47). Attempts were made to farm with Eurasian wild boars and cross-bred domestic pigs inside the controlled area in LP and MP, and to utilize them for hunting, but the animals succumbed to ASFV (16, 18, 20, 48). As mentioned in *Results*, an outbreak of ASFV involving domestic pigs and Eurasian wild boars occurred on a farm where warthogs were present in NWP in 2019, and three blood samples were received from wild boars in NWP, GP, and FSP for the present project (Table 2 and Figure 4). Since Eurasian wild boars usually succumb to ASFV infection, it was to be expected that the three blood samples tested negative for antibody to the virus, but the presence of these animals in NWP, GP, and FSP suggests that there is need to monitor the distribution and growth of populations in South Africa, particularly since the species has proved to be a highly invasive elsewhere.

The present findings confirm that there is circulation of ASFV in warthogs beyond the controlled area in South Africa (Table 2 and Figures 4, 5), but the full extent of spread of infection and the mechanisms involved remain largely undetermined. Unbeknown to the present authors, aliquots of 91 of the same GKNP sera were included in an unrelated survey with similar ASFV findings (49), but this is immaterial since the samples were used in the current study mainly to confirm the validity of the antibody test for warthog sera. Samples from extralimital warthogs were of central interest.

Warthog piglets are infected by ticks while confined to burrows during early life and develop intense viremia that in turn facilitates infection of ticks (4). Low-intensity viremia and infection of lymphatic tissues may persist for months but warthogs are not contagious for domestic pigs or each other (1, 40). There is controversy about the infectivity of warthog offal for domestic pigs, but it has recently been confirmed that low doses of virus are infective for pigs by mouth (7, 50). The majority of outbreaks of ASF recorded within the controlled area in South Africa involved known contact between domestic pigs and live warthogs, carcasses, or offal (16).

Transmission of ASFV by ticks was discovered in Spain after introduction of the virus into Europe and involved domestic pigs and *Ornithodoros (Pavlovskyella) erraticus* ticks (51). Circulation of ASFV in domestic pigs and *O. (O.) moubata* complex ticks living in crevices of poorly constructed animal shelters was documented in Malawi and probably occurs elsewhere in Africa, but has not been reported in South Africa (7, 52). *Ornithodoros* ticks generally engorge blood meals in minutes to hours and detach while animals are at rest but can be conveyed passively on their hosts between burrows or to the environs of domestic pigs to transmit infection (53–56). Transport of *Ornithodoros* ticks on live warthogs or carcasses to the environs of piggeries has been observed in the controlled area (16).

The original warthog translocations in South Africa were undertaken by officials of national and provincial parks in order to replace the Cape warthog (*P. aethiopicus aethiopicus*), formerly present in the south of the country but extinct after the rinderpest epidemic of 1896 (57, 58). Warthogs were sourced from the Hluhluwe-iMfolozi Game Reserve in KwaZulu-Natal Province considered to be free of *Ornithodoros* ticks and ASFV at the time (7, 22). Translocations were made to the ECP in 1976–1977 and from there to Rolfontein NR in NCP in 1984. From 1991 onwards, Rolfontein NR served as the source for transfers to other reserves in NCP (24, 59). The lack of antibody to ASFV in warthog sera collected in Rolfontein NR in 2016 and Addo ENP in ECP in 2019 (Figure 5) implies that the virus was not disseminated during the original warthog translocations.

When Mokala NP was established in 2007, many animals were transferred from the former Vaalbos National Park in NCP, but warthogs were not included since they were already present on the farms incorporated into the new park (Mr. J. de Klerk, Manager, Mokala NP, personal communication, 2019). Hence, the high prevalence of antibody found in Mokala NP in 2017 and 2019 implies that the virus is endemic in the region, not just in the park. This is consistent with the detection of seropositive warthogs on farms 18 and 19 (Table 2) and the histories of contact of domestic pigs with warthog tissues in the outbreaks of ASF recorded on farms in NCP and adjacent FSP in 2017 (Figure 4).

Major nature reserves have not been implicated in triggering outbreaks of ASF in domestic pigs in South Africa, but the large populations of warthogs present on private land appear to play a direct role. To what extent multiple unrecorded translocations of warthogs that accompanied the burgeoning of game ranches and private nature reserves (24, 60) promoted dispersal of ticks and ASFV is less important than the potential threat posed by continued expansion of extralimital warthog populations and possible utilization without due regard to biosafety measures. The threat is greater for small-scale pig farmers who exercise little control on potential introduction of infection (6, 61, 62) than it is for commercial producers that apply strict compartmentalization procedures.

The taxonomy of Afrotropical *Ornithodoros* ticks was recently reviewed with description of new species and type localities, but little is known about ASFV vector competence and distribution ranges for some species (63). Observations on the occurrence of *Ornithodoros* tick species and their ASFV infection status in relation to a selection of the current study sites will be presented separately.

## Data Availability Statement

The original contributions presented in the study are included in the article/supplementary material, further inquiries can be directed to the corresponding author.

## Ethics Statement

The animal study was reviewed and approved by University of Pretoria—Animal Ethics Committee. Written informed consent was obtained from the owners for the participation of their animals in this study.

## Author Contributions

AC: planned and performed the study and prepared the manuscript under supervision as part of a Ph.D. project. MS-W: provided guidance in conducting laboratory procedures and provided field assistance. HH: provided guidance in conducting laboratory procedures. LH: provided guidance in conducting laboratory procedures and analysis and interpretation of data. GK: provided regional state veterinary supervision and assistance in conducting field studies. L-MK-L and LS: provided regional state veterinary supervision and assistance in conducting field studies. PB: provided guidance and access to biobanked warthog samples. JT: provided guidance in conducting laboratory procedures. JC: co-supervised the study and provided guidance in conducting field studies. JR: suggested conducting a research project on African swine fever in South Africa and raised the funds for the research contract. RS: supervised planning and performance of the study and preparation of the manuscript. All authors contributed to critical revision of the manuscript and approved the final version to be submitted for publication.

## Funding

We acknowledge the support of NBAF Transition funds from the State of Kansas, the P20GM130448 under Award No. P20GM130448, and the Department of Homeland Security Center of Excellence for Emerging and Zoonotic Animal Diseases under Grant No. HSHQDC 16-A-B0006 to JR.

## Conflict of Interest

The authors declare that the research was conducted in the absence of any commercial or financial relationships that could be construed as a potential conflict of interest.

## Publisher's Note

All claims expressed in this article are solely those of the authors and do not necessarily represent those of their affiliated organizations, or those of the publisher, the editors and the reviewers. Any product that may be evaluated in this article, or claim that may be made by its manufacturer, is not guaranteed or endorsed by the publisher.
